# Early Changes in Myocardial Mechanics and Diastolic Function in Women During the Menopausal Transition

**DOI:** 10.3390/jcm15218595

**Published:** 2026-11-04

**Authors:** Vitaliy Koikov, Gulmira Derbissalina, Zhanar Balmukhamedova, Ayagoz Umbetzhanova, Zhanagul Bekbergenova, Natalya Zemlyanskaya, Viktor Zemlyanskiy

**Affiliations:** 1 Ministry of Health of the Republic of Kazakhstan, Astana 010000, Kazakhstan; koykov@inbox.ru; 2 Department of Family Medicine №1, “Shormanov National Medical University” NCJSC, Astana 010000, Kazakhstan; balmukhamedova.zhanar@gmail.com (Z.B.); bekbergenova.zh@amu.kz (Z.B.); zemlyanskaya.ns@gmail.com (N.Z.); 3 Interventional Radiology Department, CF “University Medical Center”, Astana 010000, Kazakhstan

**Keywords:** diastolic dysfunction, echocardiography, speckle tracking, menopause, myocardial contraction, ventricular function, left

## Abstract

**Background:** The menopausal transition is associated with cardiovascular remodeling, but it remains unclear whether regional alterations in left ventricular (LV) myocardial mechanics are present despite preserved global systolic function. This study investigated myocardial deformation and diastolic function in women during the menopausal transition, with particular attention to the effects of chronological age and cardiometabolic factors. **Methods:** This cross-sectional study included 153 women aged 45–55 years, comprising 93 perimenopausal and 60 menopausal women. Menopausal status was determined using menstrual history and hormonal assessment. Conventional echocardiography and two-dimensional speckle-tracking echocardiography were performed to assess LV structure, diastolic function, global longitudinal strain (GLS), and regional longitudinal strain. Multivariable regression models were adjusted for age, body mass index, and low-density lipoprotein cholesterol. **Results:** GLS was similar in menopausal and perimenopausal women (−19.62 ± 2.08% vs. −19.50 ± 1.96%; *p* = 0.723), whereas mean basal longitudinal strain was less negative in menopausal women (−18.14 ± 2.19% vs. −19.11 ± 2.22%; *p* = 0.009). This association persisted after multivariable adjustment (β = 1.07, 95% CI 0.27–1.88; *p* = 0.009). The largest segmental difference occurred in the basal inferior segment, which was considered an exploratory finding. Basal inferior strain was associated with LV diastolic dysfunction (OR 1.13 per 1 percentage-point shift toward less negative strain, 95% CI 1.01–1.26; *p* = 0.038). Its addition to the clinical model increased the apparent AUC from 0.619 to 0.667 (likelihood-ratio *p* = 0.035), although the difference between AUCs was not statistically significant by the paired DeLong test (*p* = 0.167). This finding should be considered exploratory and hypothesis-generating. **Conclusions:** Menopause was associated with a selective shift toward less negative basal LV longitudinal deformation and less favorable diastolic function despite preserved LVEF and GLS. In an exploratory analysis, basal inferior longitudinal strain was associated with LV diastolic dysfunction, although its apparent incremental discriminative value was modest. Regional longitudinal strain may provide complementary information on subclinical cardiac functional alterations associated with menopausal status, but its clinical utility requires validation in independent cohorts.

## 1. Introduction

Cardiovascular diseases remain one of the leading causes of death and disability worldwide [1]. Cardiovascular risk in women increases substantially during midlife, coinciding with the menopausal transition. Although chronological aging remains an important determinant of cardiovascular health, accumulating evidence suggests that reproductive aging contributes additional changes in body composition, lipid metabolism, insulin sensitivity, vascular function, and cardiac structure and function [2,3]. The menopausal transition is therefore increasingly regarded as a distinct period of cardiovascular vulnerability rather than simply a marker of advancing age. Loss of ovarian estrogen exposure has been proposed as one of the mechanisms underlying these changes, through effects on vascular function, myocardial metabolism, extracellular matrix regulation, and cardiometabolic risk factors [2,4].

Changes in cardiac structure and diastolic function may develop during this period before clinically apparent cardiovascular disease. In the CARDIA study, menopause was associated with higher E/e′ and with adverse longitudinal changes in left ventricular (LV) mass and left atrial volume after adjustment for demographic characteristics and cardiovascular risk factors [5]. Other studies have similarly linked earlier age at menopause with adverse cardiovascular outcomes, including an increased risk of heart failure and atrial fibrillation [6]. These observations are particularly relevant to women because diastolic dysfunction and heart failure with preserved ejection fraction become increasingly prevalent after menopause. However, conventional measures such as LV ejection fraction may remain normal during the early stages of myocardial remodeling and therefore provide limited information on subtle changes in myocardial function during the menopausal transition.

Speckle-tracking echocardiography provides a more sensitive assessment of myocardial mechanics by quantifying myocardial deformation. Global longitudinal strain (GLS) has been extensively investigated as a marker of subclinical LV systolic dysfunction and can identify functional abnormalities despite preserved ejection fraction [7]. Menopause has also been associated with alterations in myocardial mechanics. Sung et al. reported lower LV GLS, reduced mitral e′ velocity, greater LV mass, larger left atrial volume, and altered atrioventricular mechanics in postmenopausal women compared with premenopausal women. Lower estradiol concentrations were also associated with less favorable LV and left atrial functional indices. These findings suggest that changes in myocardial mechanics may accompany the transition to menopause before overt systolic dysfunction becomes apparent.

The interpretation of myocardial strain during menopause is nevertheless complicated by the close relationship between reproductive and chronological aging. Longitudinal deformation itself changes with age, and regional myocardial strain is not uniform across the LV. Consequently, an apparent association between menopausal status and myocardial mechanics may partly reflect age-related changes rather than an independent menopause-associated effect. This issue is particularly important when regional strain is examined. Current strain recommendations also emphasize that regional measurements have greater variability than GLS and should be interpreted in their anatomical and clinical context rather than using isolated segmental values as diagnostic thresholds [7]. Thus, studies that rely predominantly on global indices or compare premenopausal and postmenopausal women with substantial age differences may not fully distinguish the contribution of menopause from that of chronological aging.

Another unresolved question is whether early alterations in myocardial mechanics are related to the development of diastolic dysfunction. Existing studies have established associations between menopause and diastolic abnormalities [5,6] and between menopause and altered myocardial deformation [8], but these phenomena have usually been evaluated separately. Less is known about whether regional changes in LV deformation provide information on early diastolic remodeling when global systolic function and GLS are still preserved. Identification of such a pattern could help characterize an earlier stage of menopause-associated myocardial remodeling, before conventional global measures become abnormal.

Therefore, this study aimed to identify factors associated with early alterations in LV myocardial mechanics and diastolic function in women during the menopausal transition, while accounting for chronological age and major cardiometabolic factors. We specifically examined global and regional longitudinal strain together with conventional indices of LV structure and diastolic function to determine whether menopause is associated with a distinct pattern of myocardial functional remodeling despite preserved global systolic function.

## 2. Materials and Methods

### 2.1. Study Design and Participants

This observational cross-sectional study included 153 women aged 45–55 years examined between January 2020 and December 2022. Menopausal status was determined primarily on the basis of menstrual history, with serum follicle-stimulating hormone (FSH) and estradiol concentrations used as supplementary clinical information. Menopause was defined as the absence of menstruation for at least 12 consecutive months supported by a hormonal profile consistent with menopause. The study population comprised 93 perimenopausal women and 60 menopausal women. All participants were women of Kazakh ethnicity.

Women with surgical menopause, use of menopausal hormone therapy or oral contraceptives, previously diagnosed arterial hypertension, coronary artery disease, cardiomyopathy, congenital or acquired heart disease, clinically relevant arrhythmias, diabetes mellitus, severe renal, hepatic, or endocrine disease, previous chemotherapy or radiotherapy, or use of cardiotropic medications potentially affecting cardiac function were excluded.

Clinical assessment included anthropometric characteristics and cardiovascular risk factors. Laboratory testing included serum FSH and estradiol levels, glucose, creatinine, urea, total cholesterol, LDL-C, HDL-C, and triglycerides.

An exploratory secondary analysis was performed among participants with preserved global longitudinal strain (GLS < −18%) to assess whether regional myocardial deformation was associated with the presence of LV diastolic dysfunction despite preserved global systolic deformation. To address the potential confounding effect of chronological age, participants were additionally stratified into two age groups, 45–50 and 51–55 years.

Echocardiography and myocardial strain

Transthoracic echocardiography was performed using standard parasternal and apical views with simultaneous electrocardiographic recording. LV ejection fraction was calculated using the biplane Simpson method. Diastolic assessment included transmitral E and A velocities, E/A ratio, tissue Doppler e′ velocity, E/e′ ratio, and left atrial volume index (LAVI). LV diastolic function was assessed according to the 2016 recommendations of the American Society of Echocardiography and the European Association of Cardiovascular Imaging (ASE/EACVI) [9]. The assessment incorporated four key echocardiographic variables: tissue Doppler e′ velocity, average E/e′ ratio, left atrial volume index, and peak tricuspid regurgitation velocity. The corresponding abnormal thresholds were septal e′ < 7 cm/s or lateral e′ < 10 cm/s, average E/e′ > 14, left atrial volume index >34 mL/m^2^, and peak tricuspid regurgitation velocity >2.8 m/s. LV diastolic dysfunction was classified when three or more of these four parameters were abnormal; one or fewer abnormal parameters indicated normal diastolic function, whereas two abnormal parameters were considered indeterminate. Other Doppler parameters, including transmitral E and A velocities, E/A ratio, deceleration time, and isovolumetric relaxation time, were also assessed as part of the comprehensive echocardiographic evaluation.

Two-dimensional speckle-tracking echocardiography was performed using a Vivid E95 ultrasound system (GE HealthCare, Chicago, IL, USA). Offline analysis of left ventricular global and segmental longitudinal strain was performed using EchoPAC software, version 203 (GE HealthCare), with the Automated Function Imaging (AFI) technology.

Apical four-, two-, and three-chamber views were acquired at 50–90 frames/s, with at least three consecutive cardiac cycles recorded during sinus rhythm. Tracking was visually verified and manually corrected when required.

Longitudinal strain was assessed according to the standard 17-segment left ventricular model. Mean basal longitudinal strain was calculated as the average of the longitudinal strain values obtained from the six basal segments, mean mid-ventricular longitudinal strain as the average of the values obtained from the six mid-ventricular segments, and mean apical longitudinal strain as the average of the values obtained from the five apical segments. Individual segmental strain values were also evaluated, including basal inferior longitudinal strain.

All echocardiographic strain analyses were performed by the same trained operator using a standardized speckle-tracking echocardiography protocol. The operator was blinded to menopausal status and LV diastolic-function classification during strain analysis. For intraobserver reproducibility assessment, strain measurements were repeated by the same trained operator using the same echocardiographic images and standardized analysis protocol. Interobserver reproducibility was assessed independently by a second trained operator using the same stored echocardiographic images and analysis protocol. The intraobserver and interobserver assessments were performed approximately two weeks apart using two separate subsets of 20 echocardiographic examinations.

### 2.2. Statistical Analysis

Statistical analyses were performed using IBM SPSS Statistics for Windows, version 27.0 (IBM Corp., Armonk, NY, USA). Continuous variables were summarized as mean ± standard deviation or median [interquartile range], as appropriate, and categorical variables as frequencies and percentages. Between-group comparisons were performed using Welch’s independent-samples t-test for approximately normally distributed continuous variables and the Mann–Whitney U test for non-normally distributed variables. Categorical variables were compared using the chi-square test or Fisher’s exact test, as appropriate.

Mean basal longitudinal strain was considered the primary myocardial mechanics outcome. GLS, mid-ventricular and apical longitudinal strain, individual segmental strain, and indices of diastolic function were evaluated as secondary outcomes. Multivariable linear regression was used to assess associations between menopausal status and myocardial mechanics or diastolic parameters, adjusting for age, BMI, and LDL-C. Age was entered as a continuous covariate. To further address potential confounding by chronological age, additional analyses were performed within the 45–50- and 51–55-year strata, and menopause-by-age group interaction terms were tested.

Multivariable logistic regression was used to assess factors associated with LV diastolic dysfunction. Results are reported as regression coefficients (β) or odds ratios (ORs) with 95% confidence intervals (CIs), as appropriate. The incremental contribution of basal inferior longitudinal strain was evaluated by comparing nested logistic regression models using the likelihood-ratio test and by comparing the areas under the receiver operating characteristic curves (AUCs). False discovery rate correction using the Benjamini–Hochberg procedure was applied across the three regional comparisons (basal, mid-ventricular, and apical LV levels). Individual segmental strain measurements were examined as exploratory analyses and were not included in the regional-level FDR correction. Findings from individual segments were therefore considered hypothesis-generating and were interpreted cautiously.

The 95% confidence intervals for the AUCs were estimated using the DeLong method, and the difference between the correlated AUCs was assessed using a paired DeLong test. Internal bootstrap assessment was performed using 5000 resamples to estimate optimism in the apparent AUCs of the clinical and extended models. For each bootstrap sample, the models were refitted, and AUCs were calculated both in the bootstrap sample and in the original dataset; mean optimism was defined as the average difference between these AUC estimates. Optimism-corrected AUCs were calculated by subtracting the mean optimism from the apparent AUCs.

To assess intraobserver and interobserver reproducibility of myocardial deformation measurements, two separate subsets of 20 women each (10 perimenopausal and 10 menopausal participants) were evaluated. Intraobserver reproducibility was assessed by repeated measurements performed by the same operator, whereas interobserver reproducibility was assessed by comparing measurements obtained independently by two operators. Reproducibility was evaluated for global longitudinal strain (GLS), mean basal longitudinal strain, and basal inferior longitudinal strain using intraclass correlation coefficients (ICCs) with 95% confidence intervals (CIs). Bland–Altman analysis was additionally performed to assess agreement between paired measurements. Mean bias and 95% limits of agreement (LOA) were calculated as the mean difference ± 1.96 standard deviations of the differences. Differences were defined as the repeated measurement minus the initial measurement for intraobserver analysis and as the second operator’s measurement minus the first operator’s measurement for interobserver analysis.

### 2.3. Ethics

The study was approved by the Local Bioethics Committee of Astana Medical University (Protocol No. 1, 19 December 2019; subsequent approval No. 4, 29 April 2022). All participants provided written informed consent.

## 3. Results

### 3.1. Characteristics of the Study Population

A total of 153 women aged 45–55 years were included in the analysis, of whom 93 were classified as perimenopausal and 60 as menopausal. Menopausal women were older than perimenopausal women (52.97 ± 2.25 vs. 50.74 ± 2.64 years, *p* < 0.001). The groups showed the expected differences in hormonal profile. Estradiol concentrations were lower in menopausal women (59.5 [53.8–66.0] vs. 115.0 [95.0–150.0], *p* < 0.001), whereas FSH concentrations were higher (70.0 [31.8–95.5] vs. 12.0 [8.0–16.0], *p* < 0.001).

Beyond the expected difference in chronological age, no statistically significant between-group differences were observed in the principal measured cardiometabolic variables, including BMI, glucose, and lipid profile, or in the available renal biochemical markers, including creatinine and urea. LV systolic function was preserved in both groups, with no significant difference in LVEF. Menopausal women had a higher LV mass index (97.33 ± 18.07 vs. 89.04 ± 22.43 g/m^2^, *p* = 0.013) and LAVI (27.97 ± 4.78 vs. 25.97 ± 3.77 mL/m^2^, *p* = 0.007). Baseline characteristics are presented in Table 1.

### 3.2. Age-Stratified Analysis

To examine whether the observed differences were primarily attributable to chronological age, analyses were repeated within the 45–50-year and 51–55-year strata. In the 45–50-year stratum, 43 women were perimenopausal, and 13 were menopausal. In the 51–55-year stratum, 50 women were perimenopausal, and 47 were menopausal.

In women aged 45–50 years, GLS did not differ between the perimenopausal and menopausal groups (−19.39 ± 1.66% vs. −19.52 ± 2.69%, *p* = 0.869). Mean basal longitudinal strain was less negative in menopausal women, although the difference did not reach statistical significance (−17.73 ± 2.39% vs. −18.98 ± 1.90%, *p* = 0.102). The largest regional difference was observed in the basal inferior segment, where strain was substantially less negative in menopausal women (−15.38 ± 3.99% vs. −19.00 ± 4.29%, *p* = 0.010). LAVI was also higher in menopausal women (27.87 ± 3.59 vs. 24.77 ± 3.23 mL/m^2^, *p* = 0.012).

Among women aged 51–55 years, mean basal longitudinal strain was significantly less negative in the menopausal group (−18.26 ± 2.15% vs. −19.22 ± 2.47%, *p* = 0.043), whereas GLS remained similar between groups (−19.65 ± 1.91% vs. −19.60 ± 2.20%, *p* = 0.903). Basal inferior strain was again less negative in menopausal women (−18.51 ± 2.55% vs. −20.30 ± 2.57%, *p* < 0.001). Menopausal women in this age stratum also had lower e′ velocity (8.60 ± 2.79 vs. 9.72 ± 2.50 cm/s, *p* = 0.040) and higher E/e′ (9.97 ± 3.37 vs. 8.28 ± 2.66, *p* = 0.008). In an exploratory secondary analysis of the overall cohort restricted to women with preserved GLS (GLS < −18%), LV diastolic dysfunction was present in 38.3% of menopausal women and 19.4% of perimenopausal women (OR 2.57, *p* = 0.034).

LV diastolic dysfunction was present in 40.4% of menopausal women aged 51–55 years compared with 18.0% of perimenopausal women of the same age stratum (OR 3.09, *p* = 0.024). In the 45–50-year stratum, the corresponding frequencies were 38.5% and 30.2% (*p* = 0.736).

Importantly, no significant menopause-by-age group interaction was observed for basal strain (*p* = 0.733), basal inferior strain (*p* = 0.206), GLS (*p* = 0.954), e′ (*p* = 0.759), E/e′ (*p* = 0.619), LAVI (*p* = 0.168), or LV diastolic dysfunction (*p* = 0.382). Thus, there was no evidence that the association between menopausal status and myocardial mechanics or diastolic function differed materially between the two age strata (Table 2).

### 3.3. Global and Regional Left Ventricular Longitudinal Strain

Global longitudinal systolic function was preserved in both groups. GLS did not differ between perimenopausal and menopausal women (−19.50 ± 1.96% vs. −19.62 ± 2.08%, respectively; *p* = 0.723).

**Figure 1:**
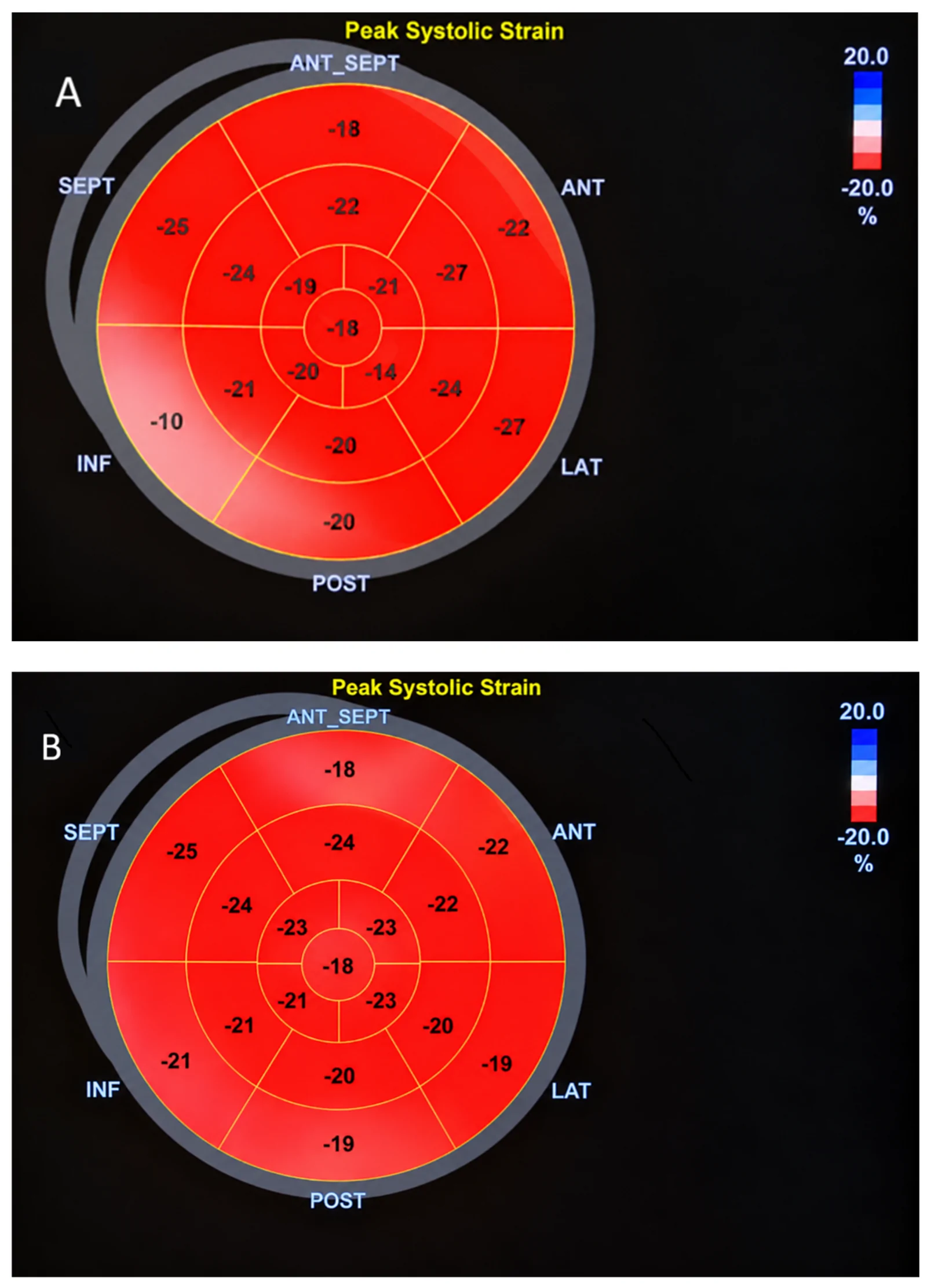
Representative bull’s-eye plots of left ventricular segmental longitudinal strain in women in menopause and perimenopause. (**A**) Menopausal woman: regional heterogeneity in longitudinal strain, including a reduced value of −10% in the inferior segment; GLS = −20.4%. (**B**) Perimenopausal woman: relatively uniform distribution of segmental longitudinal strain; GLS = −19.0%. Longitudinal strain was assessed using two-dimensional speckle-tracking echocardiography, with GLS automatically calculated using AFI.

**Figure 2:**
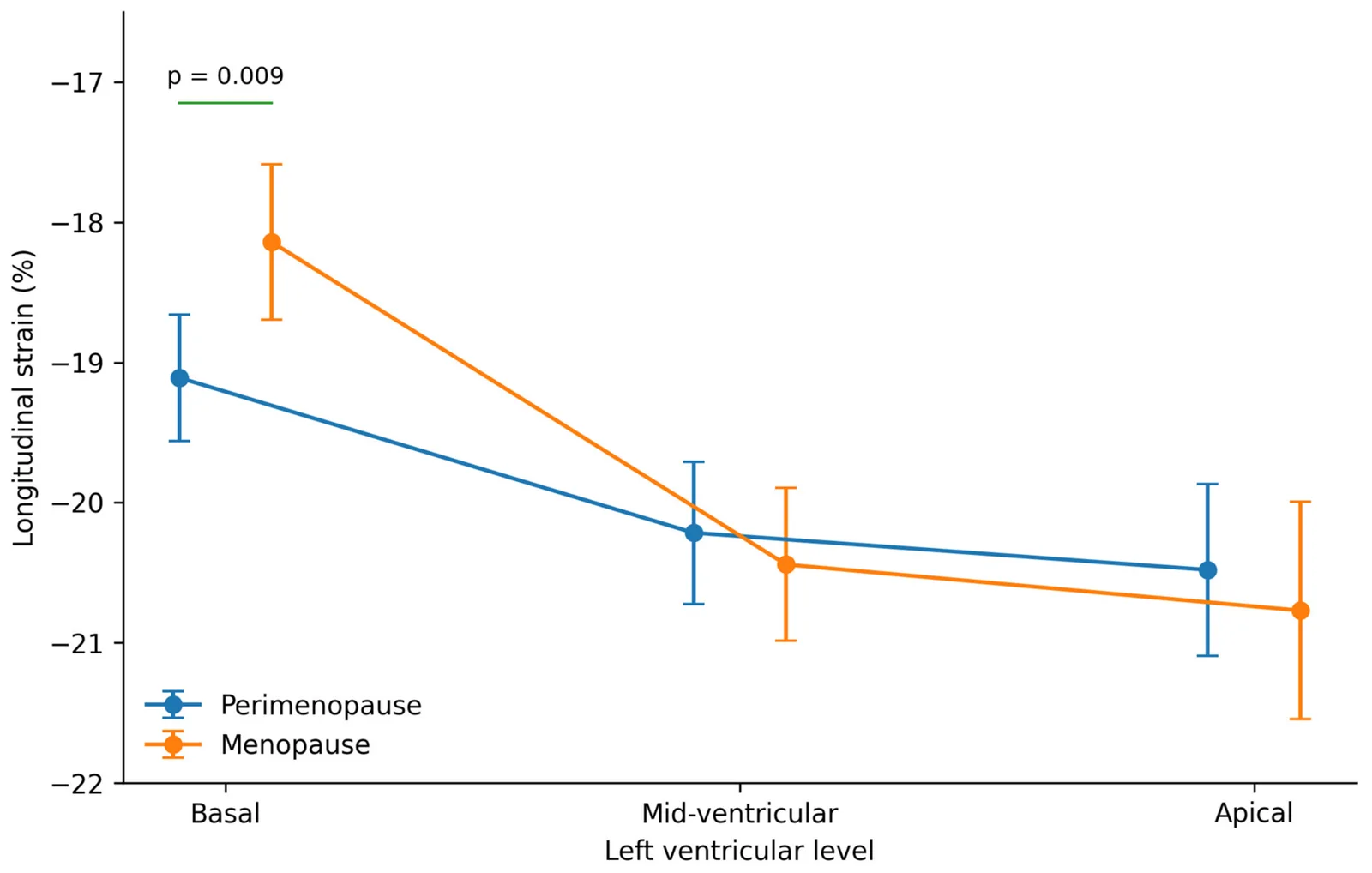
Regional left ventricular longitudinal strain according to menopausal status. Values are presented as means with 95% confidence intervals. Mean basal longitudinal strain was less negative in menopausal women than in perimenopausal women (*p* = 0.009), whereas no significant between-group differences were observed at the mid-ventricular or apical levels. After adjustment for age, BMI, and LDL-C, menopausal status remained independently associated with less negative basal longitudinal strain.

Regional analysis of longitudinal strain showed a different pattern. Mean basal longitudinal strain was less negative in menopausal women than in perimenopausal women (−18.14 ± 2.19% vs. −19.11 ± 2.22%; *p* = 0.009), whereas no significant between-group differences were observed at the mid-ventricular or apical levels (Table 3, Figure 1 and Figure 2).

After adjustment for age, BMI, and LDL-C, menopausal status remained independently associated with less negative basal longitudinal strain (β = 1.07 percentage points, 95% CI 0.27 to 1.88; *p* = 0.009). This association remained significant after correction for multiple comparisons across the basal, mid-ventricular, and apical levels (FDR-adjusted q = 0.027). No independent association with menopausal status was observed for mid-ventricular strain (β = −0.25, 95% CI −1.08 to 0.58; *p* = 0.553) or apical strain (β = −0.04, 95% CI −1.17 to 1.08; *p* = 0.939).

In the segmental analysis, the largest difference was observed in the prespecified basal inferior longitudinal strain. As individual segmental comparisons were considered exploratory, this finding was interpreted as hypothesis-generating. Basal inferior longitudinal strain was less negative in menopausal women than in perimenopausal women (−17.83 ± 3.16% vs. −19.70 ± 3.52%; *p* < 0.001), and the difference remained significant after adjustment for age, BMI, and LDL-C (β = 2.48 percentage points, 95% CI, 1.28 to 3.67; *p* < 0.001).

#### Intraobserver and Interobserver Reproducibility

Both intraobserver and interobserver analyses demonstrated high reproducibility of myocardial longitudinal strain measurements. Intraobserver ICCs were 0.960 (95% CI, 0.890–0.980) for GLS, 0.910 (95% CI, 0.790–0.960) for mean basal longitudinal strain, and 0.880 (95% CI, 0.720–0.950) for basal inferior longitudinal strain. The corresponding interobserver ICCs were 0.944 (95% CI, 0.854–0.979), 0.985 (95% CI, 0.966–0.992), and 0.928 (95% CI, 0.794–0.972), respectively.

Bland–Altman analysis demonstrated small mean differences between paired measurements. Intraobserver mean biases were −0.194, −0.182, and −0.489 percentage points for GLS, mean basal longitudinal strain, and basal inferior longitudinal strain, respectively. The corresponding interobserver biases were −0.071, −0.020, and 0.394 percentage points. The corresponding ICC estimates and 95% confidence intervals are summarized in Table 4.

### 3.4. Left Ventricular Diastolic Function

**Figure 3:**
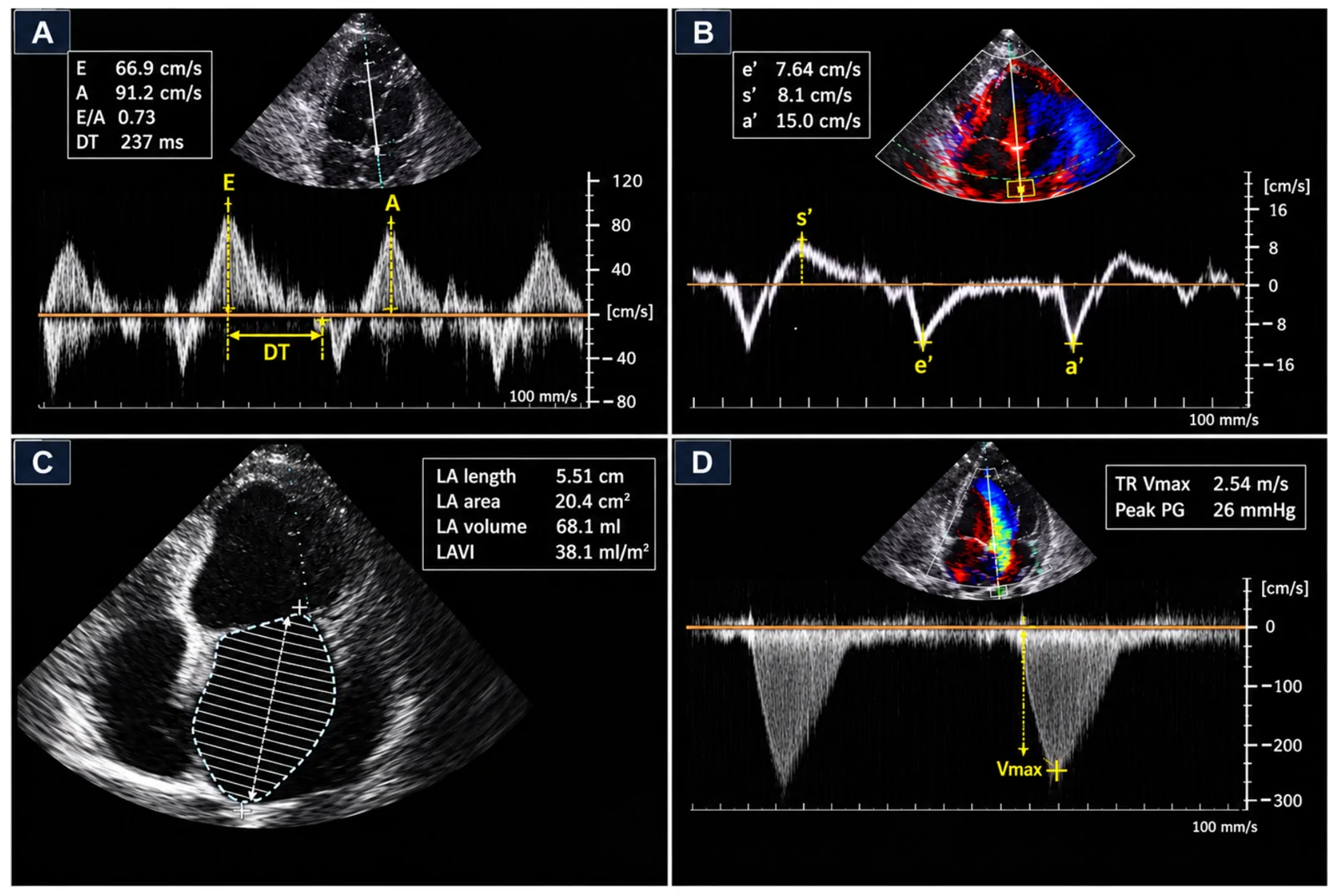
Representative echocardiographic images illustrating the assessment of left ventricular diastolic function. (**A**) Transmitral pulsed-wave Doppler showing early (E) and late (A) diastolic filling velocities (E/A ratio = 0.73; E-wave deceleration time −237 ms). (**B**) Pulsed-wave tissue Doppler imaging (PW-TDI) of the septal mitral annulus demonstrating an early diastolic myocardial velocity (e′) of 7.64 cm/s. (**C**) Left atrial volume assessment using the area-length method in the apical four-chamber view (left atrial volume index [LAVI] = 38.1 mL/m^2^). (**D**) Continuous-wave Doppler showing peak tricuspid regurgitation velocity (TR Vmax = 2.54 m/s; peak pressure gradient = 26 mmHg). The yellow crosses (+) indicate the measurement calipers used to determine TR Vmax.

Indices of LV diastolic function differed according to menopausal status. Menopausal women had lower septal e′ velocity (8.58 ± 2.68 vs. 9.54 ± 2.72 cm/s; *p* = 0.034), higher E/e′ (9.97 ± 3.57 vs. 8.65 ± 3.15; *p* = 0.021), and higher LAVI (27.97 ± 4.78 vs. 25.97 ± 3.77 mL/m^2^; *p* = 0.007). No significant between-group difference was observed for the E/A ratio (Table 5). Representative echocardiographic images illustrating the assessment of left ventricular diastolic function, including transmitral flow, mitral annular tissue Doppler imaging, left atrial volume measurement, and peak tricuspid regurgitation velocity assessment, are presented in Figure 3.

These associations were examined in multivariable models adjusted for age, BMI, and LDL-C. Menopausal status remained associated with higher E/e′ (β = 1.39, 95% CI 0.11 to 2.68; *p* = 0.034) and higher LAVI (β = 1.88 mL/m^2^, 95% CI 0.26 to 3.51; *p* = 0.023). The association with septal e′ was of borderline statistical significance (β = −0.96 cm/s, 95% CI −1.92 to −0.001; *p* = 0.050). No independent association was observed for E/A (β = 0.04, 95% CI −0.08 to 0.17; *p* = 0.528).

LV diastolic dysfunction was identified in 24 of 60 menopausal women (40.0%) and 22 of 93 perimenopausal women (23.7%; *p* = 0.049). After adjustment for age, BMI, and LDL-C, menopausal status was associated with approximately twofold higher odds of LV diastolic dysfunction, although the confidence interval included unity (OR 2.14, 95% CI 0.98 to 4.66; *p* = 0.056).

### 3.5. Association of Regional Longitudinal Strain with Left Ventricular Diastolic Dysfunction

Basal inferior longitudinal strain was associated with LV diastolic function. Less negative basal inferior strain correlated with higher E/e′ (r = 0.225, *p* = 0.005). This association remained significant after adjustment for menopausal status, age, BMI, and LDL-C (β = 0.190, *p* = 0.038).

Women with LV diastolic dysfunction had less negative basal inferior strain than women without diastolic dysfunction (−17.89 ± 3.53% vs. −19.43 ± 3.39%, *p* = 0.014). In multivariable logistic regression, basal inferior strain remained independently associated with LV diastolic dysfunction after adjustment for menopausal status, age, BMI, and LDL-C. Each 1 percentage-point shift toward less negative basal inferior strain was associated with a 13% increase in the odds of diastolic dysfunction (OR 1.13, 95% CI, 1.01–1.26; *p* = 0.038) (Table 6).

A clinical model including menopausal status, age, BMI, and LDL-C had an AUC of 0.619 (95% CI, 0.522–0.716) for LV diastolic dysfunction. The addition of basal inferior longitudinal strain increased the apparent AUC to 0.667 (95% CI, 0.571–0.762), corresponding to an absolute increase of 0.048. The addition of basal inferior longitudinal strain significantly improved model fit compared with the clinical model alone (likelihood-ratio *p* = 0.035). Because the segmental analyses were exploratory and the association between basal inferior strain and LV diastolic dysfunction was evaluated within the same cohort, this apparent improvement in discrimination should be considered potentially optimistic and hypothesis-generating rather than evidence of incremental clinical utility. In the extended model, the association with menopausal status was attenuated (OR 1.61, 95% CI, 0.71–3.67; *p* = 0.253).

Internal bootstrap assessment using 5000 resamples yielded mean optimism estimates of 0.0565 for the clinical model and 0.0553 for the extended model, corresponding to optimism-corrected AUCs of 0.563 and 0.611, respectively. Because the segmental analyses were exploratory and the basal inferior parameter was evaluated within the same dataset, the optimism-corrected estimates should be interpreted cautiously; bootstrap correction does not completely eliminate potential optimism related to the assessment of multiple segmental measurements and their subsequent evaluation in the same cohort.

Diastolic dysfunction was also examined among women with preserved global longitudinal strain (GLS < −18%). Within this subgroup, LV diastolic dysfunction was present in 38.3% of menopausal women compared with 19.4% of perimenopausal women (OR 2.57; *p* = 0.034).

## 4. Discussion

The main finding of this study was a selective alteration in basal LV longitudinal deformation in menopausal women despite preserved LVEF and GLS. Mean basal longitudinal strain was less negative after menopause, and this association persisted after adjustment for age, BMI, and LDL-C. The largest segmental difference was observed in the prespecified basal inferior segment. Menopausal status was also associated with less favorable diastolic function parameters, including higher E/e′ and LAVI and lower septal e′ velocity. Basal inferior strain was independently associated with LV diastolic dysfunction, although its apparent contribution to model discrimination was modest and exploratory.

These findings are consistent with evidence that the menopausal transition is accompanied by cardiovascular changes beyond those attributable to chronological aging alone. The American Heart Association has identified menopause as a period associated with adverse changes in cardiovascular risk factors and vascular function [2]. Cardiac remodeling has also been reported. In the CARDIA study, menopause was associated with higher E/e′ and longitudinal changes in LV mass and left atrial volume after adjustment for demographic and cardiovascular risk factors [5]. Early menopause has likewise been associated with abnormal diastolic function and adverse cardiovascular outcomes [6,10]. In our cohort, menopausal women had higher LV mass index and LAVI, while BMI, lipid profile, and LVEF were similar between groups. The persistence of the associations with E/e′ and LAVI after adjustment for age, BMI, and LDL-C supports an association between menopausal status and early changes in cardiac structure and diastolic function.

Previous STE studies have suggested that changes in myocardial deformation may become apparent before overt systolic dysfunction develops. Sung et al. reported less favorable LV longitudinal deformation together with alterations in atrial and diastolic function in postmenopausal women [8], while previous work in perimenopausal women has also demonstrated subclinical myocardial deformation abnormalities despite the absence of apparent diastolic dysfunction [11]. In contrast, GLS did not differ significantly between groups in our study, with differences becoming apparent only when longitudinal deformation was assessed regionally. More broadly, diastolic abnormalities may be detectable before overt systolic impairment in some myocardial diseases, although the temporal relationship between diastolic and systolic dysfunction varies by disease context. Impaired myocardial relaxation and increased ventricular stiffness are recognized features of diastolic dysfunction and heart failure with preserved ejection fraction [12,13,14]. In our study, LV diastolic dysfunction was also more frequent among menopausal women despite preserved GLS. However, given the cross-sectional design, these findings should not be interpreted as evidence that diastolic dysfunction necessarily precedes systolic impairment during the menopausal transition.

The distinction between statistical and clinical significance is important when interpreting these findings. The difference in mean basal strain between groups was approximately 1 percentage point and, although statistically significant, does not by itself indicate clinically established myocardial dysfunction. Regional strain values show greater biological and technical variability than GLS, and universally accepted diagnostic thresholds for individual LV regions or segments are lacking [7,15]. Similarly, the approximately 1.9 percentage-point unadjusted difference in basal inferior strain should not be interpreted as evidence of a pathological segment in individual women. Rather, these findings indicate a group-level shift toward less negative basal deformation. Their potential relevance arises from the consistency of this pattern with concomitant diastolic abnormalities rather than from crossing a predefined strain threshold.

Importantly, both intraobserver and interobserver reproducibility analyses supported the reliability of myocardial longitudinal strain measurements. High agreement was observed for GLS, mean basal longitudinal strain, and basal inferior longitudinal strain, with intraobserver ICCs ranging from 0.88 to 0.96 and interobserver ICCs ranging from 0.928 to 0.985. These findings support the technical reproducibility of both global and regional myocardial deformation measurements. Nevertheless, the Bland–Altman limits of agreement for basal inferior longitudinal strain were relatively wide and overlapped the magnitude of the observed between-group difference. Therefore, measurement variability may have contributed to the observed segmental differences, which should be interpreted cautiously.

Basal inferior longitudinal strain was prespecified as a parameter of interest and was further evaluated in relation to diastolic function, showing associations with E/e′ and LV diastolic dysfunction after adjustment for menopausal status, age, BMI, and LDL-C. Regional differences in myocardial function may reflect the anatomical and functional heterogeneity of the myocardium and differences in the susceptibility of individual myocardial regions to structural and functional stress [16]. However, the specific mechanism underlying the regional strain pattern observed in the present study cannot be established from these cross-sectional data.

Chronological age represents an important potential confounder when interpreting menopause-related myocardial and diastolic changes. In the present study, menopausal women were older than perimenopausal women; therefore, the potential contribution of age cannot be completely excluded. Age was included as a continuous covariate in the multivariable models, and age-stratified analyses were performed within the 45–50-year and 51–55-year age groups. No statistically significant menopause-by-age interactions were observed for the principal myocardial deformation and diastolic function parameters. However, these findings should not be interpreted as demonstrating that the effects of chronological aging were fully accounted for, particularly given the relatively small number of menopausal women in the 45–50-year stratum (n = 13). Accordingly, the observed differences should be interpreted as associations with menopausal status after adjustment for age rather than as evidence of an exclusively menopause-specific effect.

The association between regional deformation and diastolic function provides further context for the strain findings. Diastolic dysfunction was more frequent among menopausal women, while E/e′ and LAVI remained independently associated with menopausal status. Basal inferior strain was independently associated with diastolic dysfunction, with a 13% increase in the odds of diastolic dysfunction for each 1 percentage-point shift toward less negative deformation. In the exploratory discrimination analysis, addition of basal inferior strain increased the apparent AUC from 0.619 (95% CI, 0.522–0.716) to 0.667 (95% CI, 0.571–0.762), corresponding to an absolute increase of 0.048. Although addition of basal inferior strain significantly improved model fit (likelihood-ratio *p* = 0.035), the difference between the correlated AUCs was not statistically significant (ΔAUC = 0.047, 95% CI, −0.020 to 0.114; paired DeLong *p* = 0.167). Because the segmental analyses were exploratory and the association between basal inferior strain and LV diastolic dysfunction was evaluated within the same cohort, the apparent improvement in discrimination may be optimistic and should be considered hypothesis-generating rather than evidence of incremental clinical utility. Basal inferior strain should not be considered a stand-alone diagnostic or predictive marker on the basis of these data.

Internal bootstrap assessment further supported the presence of optimism in the apparent discrimination estimates. After correction for bootstrap-derived optimism, the AUC decreased from 0.619 to 0.563 for the clinical model and from 0.667 to 0.611 for the extended model. These findings reinforce the need for caution when interpreting the apparent discrimination observed in the study cohort. Importantly, because the segmental analyses were exploratory and the basal inferior parameter was evaluated within the same dataset, the bootstrap analysis cannot completely eliminate potential optimism related to the assessment of multiple segmental measurements and their subsequent evaluation in the same cohort. Therefore, the observed association and discrimination should be regarded as hypothesis-generating rather than confirmatory and require validation in independent cohorts.

The observed differences in diastolic function may also be related to the decline in estrogen exposure accompanying menopause. Experimental and clinical evidence suggests that estrogen may influence LV relaxation through effects on vascular function, the renin–angiotensin–aldosterone system, myocardial remodeling, and calcium handling. In postmenopausal women with pre-existing LV diastolic dysfunction, estradiol administration has been associated with improvements in selected diastolic function parameters, while mechanistic and clinical evidence supports a potential role of estrogen deficiency in the development of diastolic dysfunction [17,18,19]. However, these findings should not be interpreted as evidence that estrogen replacement prevents myocardial dysfunction, and in the present study, the cross-sectional design and absence of an intervention preclude any inference regarding a causal or therapeutic effect of estrogen.

Importantly, a normal global deformation measure did not exclude abnormalities of diastolic function in this cohort. This pattern may be relevant to the predominance of women among patients who later develop HFpEF, where abnormalities in relaxation, filling pressures, atrial function, and myocardial mechanics may occur despite preserved LVEF [20,21,22]. However, our data do not demonstrate progression to HFpEF, and such an association would require longitudinal follow-up.

Several mechanisms may contribute to cardiac changes during menopause. Declining estrogen exposure has been associated with alterations in vascular function, myocardial metabolism, extracellular matrix regulation, and cardiometabolic profile [2,4]. In addition to hormonal changes, chronological aging, vascular alterations, and metabolic and structural myocardial changes may contribute to the observed abnormalities [23]. However, myocardial fibrosis or tissue composition was not directly assessed in this study. The regional strain findings therefore cannot be considered evidence of myocardial fibrosis or of a specific underlying histopathological process.

When interpreting the present findings, a broader cardiometabolic context should also be considered. Women with established hypertension, diabetes, overt cardiovascular disease, and severe renal, hepatic, or endocrine disorders were excluded from the study. In addition, no statistically significant between-group differences were observed in the principal measured cardiometabolic variables, including BMI, glucose, and lipid profile, or in the available renal biochemical markers, including creatinine and urea. The absence of significant between-group differences in these parameters reduces the likelihood that the observed differences in myocardial deformation and diastolic function were attributable to a marked imbalance in the measured cardiometabolic factors. Nevertheless, the absence of statistically significant differences in individual variables does not exclude their potential combined or subclinical effects. Contemporary cardiovascular-kidney-metabolic concepts emphasize the interconnected contribution of metabolic, renal, and cardiovascular factors to structural and functional cardiovascular abnormalities [24]. Studies conducted in Kazakhstan have also addressed the multifactorial determinants of cardiovascular care and risk, including treatment adherence and combinations of clinical, laboratory, and procedural factors [25,26]. These considerations suggest that the observed myocardial changes should be interpreted within a broader cardiometabolic context rather than attributed to menopausal status alone.

### Strengths and Limitations

The strengths of this study include detailed echocardiographic phenotyping of women without established cardiovascular disease, diabetes, or other major conditions known to affect myocardial mechanics; assessment of menopausal status using menstrual history and hormonal measurements; and evaluation of global, regional, and segmental deformation using a standardized STE protocol. The narrow age range, age-stratified analyses, and adjustment for age as a continuous covariate were particularly important for partially disentangling menopausal status from chronological age.

The study also has limitations. Its cross-sectional design does not establish temporal or causal relationships. The sample size was modest, particularly among menopausal women aged 45–50 years (n = 13), which limited the precision of age-stratified comparisons.

Although women with established hypertension, diabetes, overt cardiovascular disease, and severe renal, hepatic, or endocrine disorders were excluded, residual cardiometabolic confounding cannot be completely excluded. No statistically significant between-group differences were observed in the principal available cardiometabolic variables, including BMI, glucose, and lipid profile, or in the available renal biochemical markers, including creatinine and urea. However, the absence of statistically significant differences in individual variables does not exclude their potential combined or subclinical effects.

Residual confounding by unmeasured hormonal factors cannot be excluded despite the restrictive eligibility criteria and multivariable adjustment. Segmental strain is subject to greater measurement variability than GLS. Although both intraobserver and interobserver reproducibility were assessed in separate subsets of 20 echocardiographic examinations each, the relatively small sample sizes and the inherent variability of individual segmental strain measurements should be acknowledged. Therefore, the observed regional differences require confirmation in larger independent cohorts. Individual segmental comparisons were exploratory rather than confirmatory. Although basal inferior strain was prespecified as a parameter of interest, its association with diastolic dysfunction and its incremental discrimination were evaluated within the same cohort, creating potential for optimism. The increase in AUC from 0.619 to 0.667 was modest and should not be interpreted as demonstrating incremental clinical utility. The discrimination analysis was not externally validated; therefore, these findings should be considered hypothesis-generating and require confirmation in an independent prospective cohort.

Because of the cross-sectional design, the present findings should be interpreted as associations rather than evidence of temporal progression or causality between menopausal status and myocardial remodeling. The observed differences in regional myocardial deformation and diastolic function cannot establish whether these abnormalities developed as a consequence of the menopausal transition or preceded it. Prospective longitudinal studies are required to determine whether changes in myocardial deformation and diastolic function emerge during the menopausal transition and whether they predict subsequent clinical dysfunction.

The study population was ethnically homogeneous, as all participants were of Kazakh ethnicity. Further studies in ethnically diverse populations would be valuable to assess the generalizability of these findings.

## 5. Conclusions

Menopause was associated with a selective shift toward less negative basal LV longitudinal deformation and less favorable diastolic indices despite preserved LVEF and GLS. These associations persisted after adjustment for chronological age, BMI, and LDL-C. Basal inferior longitudinal strain was associated with LV diastolic dysfunction in an exploratory analysis, although its apparent incremental contribution to model discrimination was modest. Regional longitudinal strain may provide complementary information on early functional cardiac remodeling during the menopausal transition, but its clinical utility requires validation in independent cohorts.

## Figures and Tables

**Table 1:** Baseline characteristics according to menopausal status.

Characteristic	Total (n = 153)	Perimenopause (n = 93)	Menopause (n = 60)	*p*-Value
Age, years	51.61 ± 2.71	50.74 ± 2.64	52.97 ± 2.25	<0.001
BMI, kg/m^2^	28.30 ± 5.14	28.02 ± 5.04	28.73 ± 5.29	0.413
Estradiol, pg/mL	88.0 [62.0–123.0]	115.0 [95.0–150.0]	59.5 [53.8–66.0]	<0.001
FSH, mIU/mL	18.0 [10.0–43.0]	12.0 [8.0–16.0]	70.0 [31.8–95.5]	<0.001
Total cholesterol, mmol/L	5.36 ± 1.08	5.43 ± 1.11	5.25 ± 1.03	0.319
LDL-C, mmol/L	3.35 ± 0.96	3.39 ± 1.06	3.29 ± 0.80	0.492
HDL-C, mmol/L	1.53 ± 0.30	1.55 ± 0.29	1.51 ± 0.32	0.388
Triglycerides, mmol/L	1.51 ± 0.60	1.48 ± 0.55	1.54 ± 0.68	0.590
Glucose, mmol/L	5.39 [4.80–5.84]	5.40 [4.93–5.85]	5.38 [4.72–5.82]	0.624
Creatinine, µmol/L	65.00 [57.90–74.20]	62.15 [56.70–71.17]	67.00 [58.68–75.70]	0.173
Urea, mmol/L	4.50 [3.70–5.50]	4.78 [3.82–5.80]	4.16 [3.60–5.20]	0.113
LVEF, %	60.24 ± 2.96	60.42 ± 3.00	59.97 ± 2.90	0.354
LV mass index, g/m^2^	92.29 ± 21.16	89.04 ± 22.43	97.33 ± 18.07	0.013
LAVI, mL/m^2^	26.75 ± 4.30	25.97 ± 3.77	27.97 ± 4.78	0.007

Data are presented as mean ± standard deviation or median [interquartile range], as appropriate. Between-group comparisons were performed using Welch’s independent-samples t-test for approximately normally distributed continuous variables and the Mann–Whitney U test for non-normally distributed continuous variables. BMI, body mass index; FSH, follicle-stimulating hormone; HDL-C, high-density lipoprotein cholesterol; LAVI, left atrial volume index; LDL-C, low-density lipoprotein cholesterol; LV, left ventricle; LVEF, left ventricular ejection fraction.

**Table 2:** Age-stratified myocardial mechanics and diastolic parameters according to menopausal status.

Parameter	45–50: Perimenopause (n = 43)	45–50: Menopause (n = 13)	*p*	51–55: Perimenopause (n = 50)	51–55: Menopause (n = 47)	*p*	*p* for Interaction
GLS, %	−19.39 ± 1.66	−19.52 ± 2.69	0.869	−19.60 ± 2.20	−19.65 ± 1.91	0.903	0.954
Basal strain, %	−18.98 ± 1.90	−17.73 ± 2.39	0.102	−19.22 ± 2.47	−18.26 ± 2.15	0.043	0.733
Mid-ventricular strain, %	−20.06 ± 1.97	−20.44 ± 1.39	0.450	−20.35 ± 2.88	−20.44 ± 2.34	0.856	0.751
Apical strain, %	−20.13 ± 2.58	−21.40 ± 3.03	0.189	−20.78 ± 3.33	−20.60 ± 3.09	0.783	0.196
Basal inferior strain, %	−19.00 ± 4.29	−15.38 ± 3.99	0.010	−20.30 ± 2.57	−18.51 ± 2.55	<0.001	0.206
e′, cm/s	9.33 ± 2.98	8.54 ± 2.30	0.324	9.72 ± 2.50	8.60 ± 2.79	0.040	0.759
E/e′	9.09 ± 3.62	9.99 ± 4.38	0.510	8.28 ± 2.66	9.97 ± 3.37	0.008	0.619
LAVI, mL/m^2^	24.77 ± 3.23	27.87 ± 3.59	0.012	27.00 ± 3.93	28.00 ± 5.10	0.282	0.168
LV diastolic dysfunction, n (%)	13 (30.2)	5 (38.5)	0.736	9 (18.0)	19 (40.4)	0.024	0.382

**Table 3:** Global and regional left ventricular longitudinal strain according to menopausal status.

Parameter	Perimenopause (n = 93)	Menopause (n = 60)	Unadjusted *p*-Value	Adjusted β	95% CI	Adjusted *p*-Value
GLS, %	−19.50 ± 1.96	−19.62 ± 2.08	0.723	−0.10	−0.91 to 0.70	0.801
Basal longitudinal strain, %	−19.11 ± 2.22	−18.14 ± 2.19	0.009	1.07	0.27 to 1.88	0.009
Mid-ventricular longitudinal strain, %	−20.22 ± 2.49	−20.44 ± 2.15	0.552	−0.25	−1.08 to 0.58	0.553
Apical longitudinal strain, %	−20.48 ± 3.01	−20.77 ± 3.06	0.566	−0.04	−1.17 to 1.08	0.939
Basal inferior longitudinal strain, %	−19.70 ± 3.52	−17.83 ± 3.16	<0.001	2.48	1.28 to 3.67	<0.001

Data are presented as mean ± standard deviation. Adjusted β coefficients were obtained from multivariable linear regression models including menopausal status, age, BMI, and LDL-C. A positive β for longitudinal strain indicates a shift toward less negative deformation. The association between menopausal status and mean basal longitudinal strain remained significant after Benjamini–Hochberg correction across the basal, mid-ventricular, and apical levels (FDR-adjusted q = 0.027). BMI, body mass index; CI, confidence interval; GLS, global longitudinal strain; LDL-C, low-density lipoprotein cholesterol.

**Table 4:** Intraobserver and interobserver reproducibility of myocardial longitudinal strain measurements.

Parameter	Intraobserver ICC (95% CI)	Interobserver ICC (95% CI)
GLS	0.960 (0.890–0.980)	0.944 (0.854–0.979)
Mean basal longitudinal strain	0.910 (0.790–0.960)	0.985 (0.966–0.992)
Basal inferior longitudinal strain	0.880 (0.720–0.950)	0.928 (0.794–0.972)

ICC, intraclass correlation coefficient; CI, confidence interval; GLS, global longitudinal strain. Intraobserver and interobserver reproducibility were assessed in two separate subsets of 20 examinations each.

**Table 5:** Left ventricular diastolic function according to menopausal status.

Parameter	Perimenopause (n = 93)	Menopause (n = 60)	Unadjusted *p*-Value	Adjusted β/OR	95% CI	Adjusted *p*-Value
e′, cm/s	9.54 ± 2.72	8.58 ± 2.68	0.034	−0.96	−1.92 to −0.001	0.050
E/e′	8.65 ± 3.15	9.97 ± 3.57	0.021	1.39	0.11 to 2.68	0.034
LAVI, mL/m^2^	25.97 ± 3.77	27.97 ± 4.78	0.007	1.88	0.26 to 3.51	0.023
E/A	1.00 ± 0.36	1.01 ± 0.34	0.971	0.04	−0.08 to 0.17	0.528
LV diastolic dysfunction, n (%)	22 (23.7)	24 (40.0)	0.049	OR 2.14	0.98 to 4.66	0.056

Continuous data are presented as mean ± standard deviation and categorical data as n (%). Adjusted β coefficients were obtained from multivariable linear regression models, and the adjusted OR for LV diastolic dysfunction was obtained from multivariable logistic regression. All models included menopausal status, age, BMI, and LDL-C. CI, confidence interval; LAVI, left atrial volume index; LV, left ventricle; OR, odds ratio.

**Table 6:** Multivariable logistic regression analysis of factors associated with left ventricular diastolic dysfunction.

Predictor	OR	95% CI	*p*-Value
Menopausal status	1.61	0.71 to 3.67	0.253
Basal inferior longitudinal strain, per 1 percentage point	1.13	1.01 to 1.26	0.038
Age, per year	1.05	0.91 to 1.23	0.492
BMI, per kg/m^2^	0.97	0.91 to 1.04	0.416
LDL-C	0.96	0.66 to 1.41	0.844

The model included menopausal status, basal inferior longitudinal strain, age, BMI, and LDL-C. For longitudinal strain, a higher value indicates less negative deformation. OR, odds ratio; CI, confidence interval; BMI, body mass index; LDL-C, low-density lipoprotein cholesterol.

## Data Availability

The data presented in this study are available on request from the corresponding author due to privacy and ethical restrictions.

## Abbreviations

The following abbreviations are used in this manuscript:

AFI	Automated Function Imaging
BMI	Body mass index
CI	Confidence interval
E/A	Ratio of early to late transmitral flow velocity
E/e′	Ratio of early transmitral flow velocity to early diastolic mitral annular velocity
FDR	False discovery rate
FSH	Follicle-stimulating hormone
GLS	Global longitudinal strain
HDL-C	High-density lipoprotein cholesterol
HFpEF	Heart failure with preserved ejection fraction
LAVI	Left atrial volume index
LDL-C	Low-density lipoprotein cholesterol
LV	Left ventricular
LVEF	Left ventricular ejection fraction
LVMI	Left ventricular mass index
OR	Odds ratio
ROC	Receiver operating characteristic
STE	Speckle-tracking echocardiography
